# Genetic Determinants of Visit-to-Visit Lipid Variability: Genome-Wide Association Study in Statin-Naïve Korean Population

**DOI:** 10.3389/fcvm.2022.811657

**Published:** 2022-01-31

**Authors:** Jun-Bean Park, Eunsoon Shin, Jong-Eun Lee, Seung Jae Lee, Heesun Lee, Su-Yeon Choi, Eun Kyung Choe, Seung Ho Choi, Hyo Eun Park

**Affiliations:** ^1^Division of Cardiology, Department of Internal Medicine, Seoul National University Hospital, Seoul, South Korea; ^2^DNA Link, Inc., Seoul, South Korea; ^3^Division of Cardiology, Department of Internal Medicine, Healthcare System Gangnam Center, Seoul National University Hospital, Seoul, South Korea; ^4^Department of Surgery, Healthcare System Gangnam Center, Seoul National University Hospital, Seoul, South Korea; ^5^Division of Pulmonology, Department of Internal Medicine, Healthcare System Gangnam Center, Seoul National University Hospital, Seoul, South Korea

**Keywords:** cholesterol variability, coronary artery calcium, coronary artery stenosis, genome wide association study, Apo A5

## Abstract

**Background and Aim:**

There is a growing evidence that fluctuation in lipid profiles is important in cardiovascular outcomes. We aimed to identify single nucleotide polymorphism (SNP) variants associated with low-density lipoprotein-cholesterol (LDL-C) and high-density lipoprotein-cholesterol (HDL-C) variability in statin-naïve Korean subjects and evaluate their associations with coronary atherosclerosis.

**Methods:**

In statin-naïve subjects from Gene-Environment of Interaction and phenotype cohort, we performed genome-wide association studies of lipid variability; the discovery (first) and replication (second) sets included 4,287 and 1,086 subjects, respectively. Coronary artery calcium (CAC) score and degree of coronary artery stenosis were used as outcome measures. Cholesterol variability was determined by standard deviation and average successive variability, and significant coronary atherosclerosis was defined as CAC score ≥400 or coronary stenosis ≥70%.

**Results:**

Mean HDL-C and LDL-C level were 54 ± 12 and 123 ± 30 mg/dL in the first set and 53 ± 12 and 126 ± 29 mg/dL in the second set. *APOA5* rs662799 and *APOA5* rs2266788 were associated with LDL-C variability and *PXDNL* rs80056520, *ALDH2* rs671, *HECTD4* rs2074356, and *CETP* rs2303790 were SNPs associated for HDL-C variability. *APOA5* rs662799 passed Bonferroni correction with *p*-value of 1.789 × 10^−9^. Among the SNPs associated with cholesterol variability, rs80056520 and rs2266788 variants were associated with CACS ≥400 and coronary stenosis ≥70% and rs662799 variant was associated with coronary stenosis ≥70%.

**Conclusion:**

Two SNPs associated with LDL-C variability (*APOA5* rs662799 and rs2266788) and one SNP associated with HDL-C variability (*PXDNL* rs80056520) were significantly associated with advanced coronary artery stenosis. Combining GWAS results with imaging parameters, our study may provide a deeper understanding of underlying pathogenic basis of the link between lipid variability and coronary atherosclerosis.

## Introduction

An increased level of serum cholesterol, and more specifically, low-density lipoprotein cholesterol (LDL-C), is a well-established risk factor in the development of atherosclerosis and associated cardiovascular diseases (CVDs) (1). It is also a very common disorder, affecting 17.6% in Korean population (2), and its prevalence is continuously increasing worldwide. Besides high total cholesterol (TC) and LDL-C levels, a low high-density lipoprotein-cholesterol (HDL-C) level is a well-known risk factor of atherosclerotic CVDs (ASCVDs) (3). Several studies have consistently shown that high LDL-C and low HDL-C levels result in a deterioration in cardiovascular outcomes (4–6). Closely monitoring and effectively achieving the target cholesterol levels is thus clinically important in both primary and secondary preventions.

Recent studies show growing evidence that fluctuations in traditional cardiovascular risk factors are significantly associated with clinical outcomes, together with the high average level. Over the past decade, associations of blood pressure (BP) variability with CVD risk and outcomes have become an increasingly common finding in the cardiovascular literature (7, 8). The surge of research interest in BP variability has carried over into other cardiovascular risk factors, and the representative one is cholesterol. A higher intra-individual variability of lipid measures has also been shown to be associated with higher occurrence of adverse cardiovascular events, in patients with or without ASCVD (9–11). A *post-hoc* analysis from the Treating to New Target trial showed that in patients with stable coronary artery disease (CAD), visit-to-visit LDL-C variability was a powerful and independent predictor of any coronary event, any CVD event, death, myocardial infarction (MI), or stroke, independent of achieved LDL-C levels (10). In patients without history of MI and stroke, high variability of TC levels was associated with an 8% higher risk of MI and a 26% higher risk of all-cause mortality during a median follow up of 8.3 years (11). However, one major limitation addressed in these previous studies was the lack of consideration in the use of lipid-lowering agents, which might be a serious confounding factors influencing both mediator (i.e., lipid variability) and outcome variable (i.e., ASCVD risk). This can also introduce other confounders, such as treatment adherence or change in statin dose, making it difficult to prove causality in trying to analyze the effect of lipid variability.

Beside this problem of potential confounding factor, the causality for the association between lipid variability and ASCVD risk remains unclear, for which critical reasons are the difficulty in conducting a randomized controlled trial to address this question and no obvious pathophysiological and biological rationale yet. Elucidating the genetic variants that contribute to the development of lipid variability may be helpful in understanding pathophysiological mechanisms underlying the link between lipid variability and ASCVD risk. However, the genetic variants that determine visit-to-visit lipid variability has not been established in association with clinical or subclinical ASCVD.

Despite numerous studies correlating lipid variability with clinical outcomes, there is only scarce data on the effect of lipid variability on coronary atherosclerosis. Since coronary atherosclerosis is a crucial pathophysiologic mechanism involved in ASCVD, especially CAD, identifying genetic determinants of coronary atherosclerosis can provide mechanistic insights into their role in the pathogenesis of ASCVD. Imaging surrogate markers of coronary atherosclerosis, such as coronary artery calcium (CAC) and coronary artery lumen stenosis, are reported to be superior to clinical risk factors for the prediction of long-term risk of ASCVD (12). Furthermore, considering that imaging modalities can assess the pathological process of the vascular wall itself, it is not surprising that imaging markers have advantages over classic blood markers which can merely reflect a part of complex pathways leading to coronary atherosclerosis (13). Hence, in daily practice, CAC score (CACS) and degree of coronary artery luminal stenosis remain the dominant measure of calcified atherosclerosis burden and CAD severity, respectively, and both are used to guide patient management.

In this study, we aimed to investigate genetic determinants of visit-to-visit variability in LDL-C and HDL-C in statin-naïve general population using the genome-wide association studies (GWAS) approach. To discern clinical significance of single nucleotide polymorphism (SNP) variants identified by GWAS, we also evaluated SNP variants in association with CACS and coronary artery stenosis.

## Methods

### Study Subjects

This study was conducted as a *post-hoc* analysis of the previously reported Gene-Environment of Interaction and phenotype (GENIE) cohort, in which blood samples from 17,455 people (9,396 men and 8,059 women) were collected during a routine health check-up program at Seoul National University Hospital Healthcare System Gangnam Center (14). This large population-based cohort provides comprehensive data sets of genetic and phenotypic information, such as SNP, lifestyle, medical history, and biochemical biomarkers related to non-communicable diseases. Briefly, all subjects completed self-administered questionnaire, which included information on lifestyle factors, past medical history, comorbidities, and medications. The details of the cohort have been described previously (14). In the present study, subjects were eligible for study inclusion if they had taken blood tests for more than three times, thus allowing the analysis of lipid variability. According to this inclusion criteria, 6,276 subjects were eligible and screened for medical history and status among 10,349 subjects who agreed to provide genetic information. We excluded subjects at baseline and during follow-up when they were treated with lipid-lowering medications, leaving 5,373 statin-naïve subjects for the GWAS analysis. Among these subjects, GWAS was performed on two sets: first (discovery) and second (validation) set each consisted of 4,287 and 1,086 unrelated subjects, respectively, who passed quality control. Among a total study population of 5,373 subjects, CT scans obtained after lipid measurements were available in 1,801 subjects.

The Institutional Review Board (IRB) of the Seoul National University Hospital approved the storage of bio-specimens with written informed consent (IRB number H-1103-127-357). We retrospectively collected the clinical and genetic parameters, for which the IRB approved this study protocol (IRB number H-1803-081-930) and waived additional informed consent. This study was also performed in accordance with the Declaration of Helsinki.

### Measurement of Anthropometric and Laboratory Data

Anthropometric measurements and blood tests were conducted as part of a general screening evaluation. Anthropometric parameters, including body weight, height, and waist circumference, were measured on the day of the examination, and body mass index (BMI) was calculated according to the formula: BMI = weight (kg)/height (m^2^). Waist circumference was measured at the midpoint between the lower costal margin and the iliac crest by a well-trained nurse. The blood samples were taken after 12 h fasting; complete blood cell counts, TC, triglyceride (TG), HDL-C, fasting blood glucose, glycated hemoglobin, blood urea nitrogen, creatinine, aspartate aminotransferase, alanine aminotransferase, and gamma-glutamyl transpeptidase were measured. In subjects with a TG <400 mg/dL, LDL-C was calculated using the Friedewald equation: LDL-C=TC–(HDL-C+TG/5) (15). In subjects with a TG ≥400 mg/dL, the measured LDL-C was used for analysis.

### Visit-to-Visit Lipid Variability

Among the four lipid traits, including TC, TG, HDL-C, and LDL-C, we evaluated HDL-C and LDL-C variability in this study. Visit-to-visit lipid variability was defined as variability in HDL-C and LDL-C as measured at least three times during the health examinations, and four measurements of variability were used (8, 16, 17): (1) standard deviation (SD), (2) average successive variability (ASV), (3) coefficient of variation (CV), and (4) variation independent of mean (VIM). The CV was calculated by SD/mean, and VIM was calculated as 100 × SD/mean^β^, where β is the regression coefficient based on natural logarithm of SD on natural logarithm of mean. This uncorrected VIM was corrected by using the formula: [VIM uncorrected×(mean of CV)]/(mean of VIM uncorrected). High and low lipid variability was defined by parameters of LDL-C or HDL-C variability above or below the median value, respectively.

### Genotyping and Quality Control

The genomic DNA was extracted from peripheral blood leukocytes of the participants using QuickGene DNA blood kit L with QuickGene-610 L equipment (KURABO, Osaka, Japan) according to standard protocols. Hybridization on Affymetrix Axiom KORV1.0–96 array Axiom2.0 Reagent Kit (Affymetrix, Santa Clara, CA, USA) was used according to the manufacturer's protocol. Approximately 200 ng of genomic DNA was amplified and randomly fragmented into 25–125 base pair (bp) fragments. The initial amplification of Genomic DNA was performed in 40 μl reaction volume, containing 20 μl volume of genomic DNA at a concentration of 10 ng/μl and 20 μl of Denaturation Master Mix. The reaction of initial amplification was performed for 10 min at room temperature. Subsequently, the incubated products were amplified with 130 μl of Axiom 2.0 Neutral Soln, 225 μl of Axiom 2.0 Amp Soln, and 5 μl of Axiom 2.0 Amp Enzyme. The amplification reactions were conducted for 23 ± 1 h at 37°C. The amplification of products was performed under optimized reaction to amplify fragments between 200 and 1,100 bp. A fragmentation step then reduced the amplified products to segments of ~25–125 bp, which were then end-labeled using biotinylated nucleotides. Following hybridization, the bound target was washed under stringent conditions to remove non-specific background to minimize background noise caused by random ligation events. Each polymorphic nucleotide was queried via a multi-color ligation event conducted on the array surface. After ligation, the arrays were stained and imaged on the GeneTitan MC Instrument (Affymetrix, Santa Clara, CA, USA). The image was analyzed using Genotyping Console Software (Affymetrix, Santa Clara, CA, USA). Genotype data were produced using K-CHIP available through the K-CHIP consortium. The K-CHIP was designed by the Center for Genome Science, Korea National Institute of Health, Korea (4845–301, 3000–3031).

### Analysis of Coronary Atherosclerosis on Computed Tomography

Coronary calcium scoring computed tomography (CT) and coronary CT angiography (CCTA) were acquired using a 16-slice scanner (Somatom Sensation 16; Siemens Medical Solutions, Forchheim, Germany) or a 256-slice multidetector CT scanner (Brilliance iCT 256; Philips Medical Systems, Cleveland, OH, USA), respectively. With regard to the time sequence between serial lipid measurements and imaging study, the initial lipid measurement preceded CT scans, while the second and further lipid measurements were performed before, at the time of, or after CT evaluation. A standard scanning protocol was used: with a tube voltage of 120 kV, 170 effective mA, and 0.37 ms rotation time for the 16-slice CT and with 128 × 0.625 mm section collimation, 0.27 ms rotation time, 120 kV tube voltage and 800 mA tube current for the 256-slice multidetector CT. All scans were performed with electrocardiogram-gated dose modulation. The CACS was calculated using commercially available CT software (Rapidia 2.8; INFINITT, Seoul, Korea) and the Agatston method (18). Significant coronary atherosclerosis was defined as a CACS ≥400 on coronary calcium scoring CT or presence of a plaque associated with ≥70% stenosis in any of the major epicardial coronary arteries on CCTA.

### Statistical Analysis

The SNP determined to have genome-wide significance after Bonferroni correction in the first set was screened using the Affymetrix Axiom KORV1.0–96 array. Statistical analyses were performed using the PLINK version 1.9 (https://www.cog-genomics.org/plink2) and SAS software (SAS Institute, Cary, NC, USA). A Manhattan plot of –log_10_P was generated using Haploview (http://www.broadinstitute.org/haploview). Regional association plots were created using the LocusZoom (http://locuszoom.org).

The baseline characteristics of the study population were presented as mean ± SD for continuous variables and number with proportion for categorical variables, respectively. Multivariate linear regression adjusted for age and sex was used to determine the effect of SNPs on HDL-and LDL-cholesterol variability indices, which were treated as continuous variables. For the analysis examining the association of the GWAS-identified SNPs with coronary atherosclerosis, we also used multivariate linear regression adjusted for age and sex.

## Results

The baseline characteristics of the study population are shown in Table 1. Briefly, the mean age of the overall study population was 52 ± 9 years and 3,096 (57.6%) were male. Among 5,373 study subjects, 639 (11.9%) subjects had hypertension, 124 (2.3%) subjects had diabetes, and 2,355 (43.8%) subjects had smoking history. Mean LDL-C and HDL-C levels in the entire study population were 124 ± 30 and 54 ± 12 mg/dL, respectively. Variability parameters of LDL-C and HDL-C are shown in Table 1.

**Table 1:** Baseline characteristics of the study population.

**Parameters**	**Combined set (*n* = 5,373)**	**First set (*n* = 4,287)**	**Second set (*n* = 1,086)**	*P*-value^*^
Age, yrs	52 ± 9	52 ± 9	52 ± 9	0.672
Male gender, *n* (%)	3,096 (57.6%)	2,422 (56.5%)	674 (62.1%)	<0.001
**Comorbidities**, ***n*** **(%)**				
HTN_on medication	639 (11.9%)	508 (11.8%)	131 (12.1%)	0.834
DM on medication	124 (2.3%)	99 (2.3%)	25 (2.3%)	1.000
Smoking				0.575
Never	2,328 (43.3%)	1,869 (43.6%)	459 (42.3%)	
Ex-smoker	1,324 (24.6%)	1,048 (24.4%)	276 (25.4%)	
Current smoker	1,031 (19.2%)	807 (18.8%)	224 (20.6%)	
SBP, mmHg	115 ± 15	115 ± 15	116 ± 15	0.071
DBP, mmHg	76 ± 11	76 ± 12	77 ± 11	0.025
BMI, kg/m^2^	22.9 ± 3.9	22.8 ± 3.8	23.2 ± 3.9	0.002
WC, cm	82 ± 12	82 ± 12	83 ± 12	<0.001
Hb, g/dL	14.4 ± 1.4	14.4 ± 1.4	14.7 ± 1.5	<0.001
Glucose, mg/dL	99 ± 16	98 ± 16	100 ± 19	0.005
HbA1C, %	5.7 ± 0.5	5.6 ± 0.5	5.7 ± 0.6	<0.001
BUN, mg/dL	14.0 ± 3.5	14.3 ± 3.5	13.0 ± 3.3	<0.001
Cr, mg/dL	0.83 ± 0.17	0.82 ± 0.17	0.84 ± 0.17	0.002
Total cholesterol, mg/dL	197 ± 33	196 ± 33	201 ± 33	<0.001
Triglyceride, mg/dL	109 ± 68	108 ± 68	110 ± 69	0.529
HDL-cholesterol, mg/dL	54 ± 12	54 ± 12	53 ± 12	0.186
LDL-cholesterol, mg/dL	124 ± 30	123 ± 30	126 ± 29	0.003
hsCRP, mg/dL	0.46 ± 1.54	0.47 ± 1.56	0.44 ± 1.48	0.582
**LDL variability**				
SD, mg/dL	14.9 ± 8.7	14.7 ± 8.5	15.9 ± 9.2	<0.001
CV,%	12.4 ± 6.9	12.3 ± 6.7	13.0 ± 7.3	0.002
ASV	16.9 ± 10.3	16.7 ± 10.1	17.9 ± 10.9	0.002
VIM,%	30.3 ± 16.7	29.9 ± 16.4	31.9 ± 17.7	0.001
**HDL variability**				
SD, mg/dL	4.8 ± 2.6	4.8 ± 2.6	4.9 ± 2.5	0.851
CV,%	8.9 ± 4.0	8.9 ± 4.0	9.0 ± 4.0	0.248
ASV	5.6 ± 3.2	5.6 ± 3.2	5.6 ± 3.1	0.749
VIM,%	4.2 ± 1.9	4.2 ± 1.9	4.3 ± 1.9	0.179
Mean number of measurements	5 ± 2	5 ± 2	5 ± 2	0.456
Mean follow up period for measurements, in months	60 ± 21	59 ± 19	69 ± 25	<0.001

*ASV, average successive variability; BUN, blood urea nitrogen; BMI, body mass index; CV, coefficient of variation; Cr, creatinine; DM, diabetes mellitus; DBP, diastolic blood pressure; Hb, hemoglobin; HDL, high density lipoprotein; hsCRP, high-sensitivity C reactive protein; HTN, hypertension; LDL, low-density lipoprotein; SD, standard deviation; SBP, systolic blood pressure; VIM, variation independent of mean*.

* *p-value for comparison between first and second sets*.

The clinical characteristics and laboratory measurements of two sets are given in Table 1. The mean age and comorbidities including hypertension, diabetes, and smoking history did not significantly differ between the two sets (*p* = 0.672 for age, *p* = 0.834 for hypertension, *p* = 1.000 for diabetes, and *p* = 0.575 for smoking). Four parameters of HDL-C variability did not significantly differ either (*p* = 0.851 for SD, *p* = 0.749 for ASV, *p* = 0.248 for CV, and *p* = 0.179 for VIM), while all indices of LDL-C variability were significantly lower in the first set (p<0.001 for SD, *p* = 0.002 for ASV, *p* = 0.002 for CV, and *p* = 0.001 for VIM of LDL-C variability). Compared to the second set, BMI and mean LDL-C were significantly lower in the first set (*p* = 0.002 for BMI and *p* = 0.003 for mean LDL-C).

### SNPs Associated With Visit-to-Visit Lipid Variability

Table 2 summarizes the six identified SNPs that significantly affected the variability of HDL-C and LDL-C in the first set. Specifically, *APOA5* (apolipoprotein A5) rs662799 and *APOA5* rs2266788 were significantly related with LDL-C variability as measured by SD and ASV. *PXDNL* (peroxidasin-like protein) rs80056520, *ALDH2* (Aldehyde Dehydrogenase 2) rs671, *HECTD4* (HECT Domain E3 Ubiquitin Protein Ligase 4) rs2074356, and *CETP* (cholesteryl ester transfer protein) rs2303790 were significantly associated with HDL-C variability defined as SD and ASV.

**Table 2:** SNPs associated with HDL-cholesterol and LDL-cholesterol variabilities.

**rs_number**	**Chromosome**	**Position**	**Gene**	**Minor**	**Major**	**BETA**	**SE**	***P***
**Variability by ASV**								
**LDL cholesterol variability**								
rs662799	11	116,663,707	APOA5	G	A	1.236	0.2403	2.772 × 10^−7^
rs2266788	11	116,660,686	APOA5	G	A	0.780	0.2647	3.231 × 10^−3^
**HDL cholesterol variability**								
rs80056520	8	52,466,803	PXDNL	A	G	0.256	0.1151	2.599 × 10^−2^
rs671	12	112,241,766	ALDH2	A	G	−0.354	0.0919	1.171 × 10^−4^
rs2074356	12	112,645,401	HECTD4	A	G	−0.318	0.0953	8.695 × 10^−4^
rs2303790	16	57,017,292	CETP	G	A	0.637	0.1671	1.399 × 10^−4^
**Variability by SD**								
**LDL cholesterol variability**								
rs662799	11	116,663,707	APOA5	G	A	1.224	0.2031	1.789 × 10^−9^
rs2266788	11	116,660,686	APOA5	G	A	0.905	0.2238	5.342 × 10^−5^
**HDL cholesterol variability**								
rs80056520	8	52,466,803	PXDNL	A	G	0.194	0.0924	3.618 × 10^−2^
rs671	12	112,241,766	ALDH2	A	G	−0.411	0.0736	2.466 × 10^−8^
rs2074356	12	112,645,401	HECTD4	A	G	−0.381	0.0766	6.619 × 10^−7^
rs2303790	16	57,017,292	CETP	G	A	0.681	0.134	3.918 × 10^−7^

*ASV, average successive variability; HDL, high density lipoprotein; LDL, low-density lipoprotein; SD, standard deviation*.

**Figure 1:**
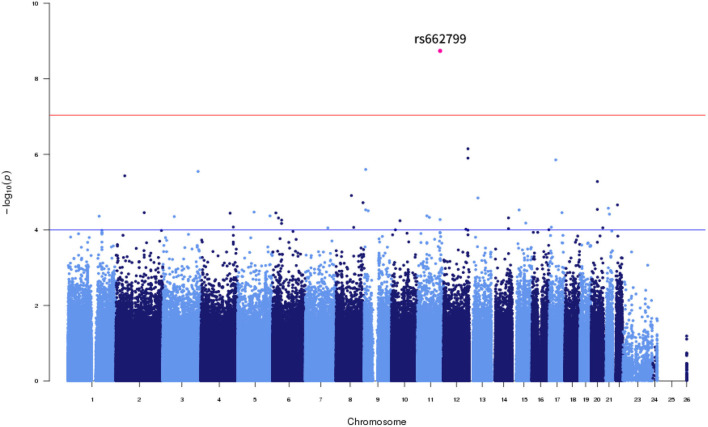
Manhattan Plot for LDL cholesterol variability by SD.

**Figure 2:**
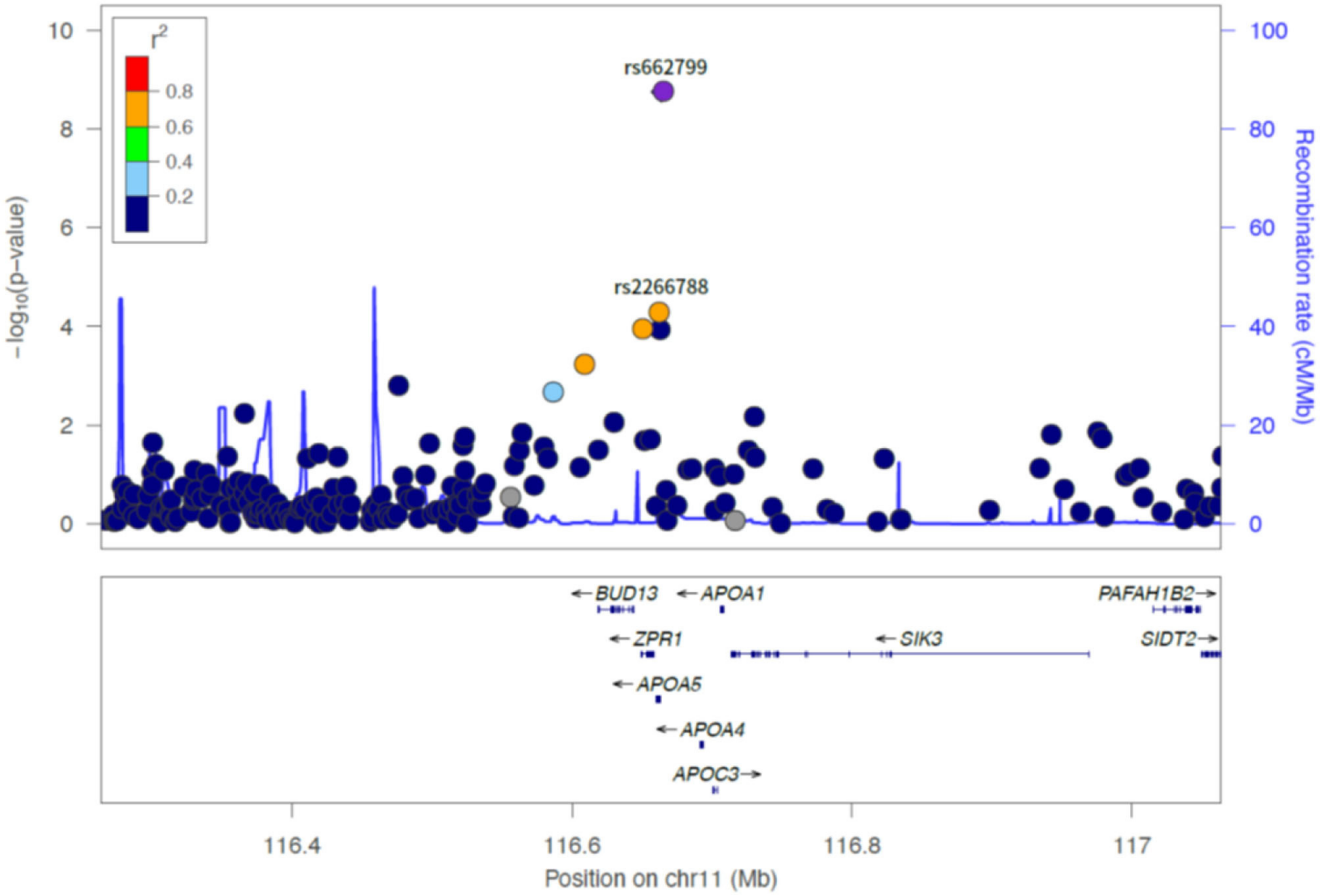
LocusZoom plot showing the region associated with LDL cholesterol variability near rs662799 (APOA5 gene).

Of the six SNPs, the *APOA5* variant rs662799 was significantly associated for LDL-C variability in the first set, which also passed Bonferroni correction (*p* = 1.789 × 10^−9^, *p* = 1.061 × 10^−8^, *p* = 2.772 × 10^−7^, and *p* = 4.632 × 10^−9^ for LDL-C variability by SD, CV, ASV, and VIM, respectively, Figures 1, 2). The *APOA5* variant rs2266788 was significantly related with LDL-C variability (*p* = 5.342 × 10^−5^, *p* = 2.289 × 10^−5^, *p* = 3.546 × 10^−3^, *p* = 2.106 × 10^−5^ for LDL-C variability by SD, CV, ASV, and VIM, respectively). The results were similar in the second set; the *APOA5* variant rs662799 and the *APOA5* variant rs2266788 were SNPs significantly related with LDL-C variability (*p* = 1.211 × 10^−3^, *p* = 1.707 × 10^−3^, *p* = 8.268 × 10^−3^ and 1.388 × 10^−3^ for LDL-C variability by SD, CV, ASV, and VIM, respectively).

The *ALDH2* variant rs671 was significantly associated with HDL-C variability, with *p*-value passing Bonferroni correction (*p* = 2.466 × 10^−8^ in the discovery set for HDL-C variability by SD). *PXDNL* variant rs80056520, *HECTD4* variant rs2074356, and *CETP* variant rs2303790 were significant determinants of HDL-C variability (*p* = 3.618 × 10^−2^, 1.03 × 10^−2^, *p* = 2.599 × 10^−2^, and *p* = 9.327 × 10^−3^ for *PXDNL* variant rs80056520 by SD, CV, ASV, and VIM, *p* = 6.619 × 10^−7^ and *p* = 8.695 × 10^−4^ for *HECTD4* variant rs2074356 by SD and ASV, *p* = 3.918 × 10^−7^ and *p* = 1.399 × 10^−4^ for *CETP* variant rs2303790 by SD and ASV, respectively). From the second set, *ALDH2* rs671, *PXDNL* rs80056520, *HECTD4* rs2074356, and *CETP* rs2303790 variants were significant variants associated with HDL-C variability (*p* = 2.102 × 10^−3^ and *p* = 1.135 × 10^−3^ for *ALDH2* variant rs671 by SD and ASV, *p* = 4.637 × 10^−7^, *p* = 6.262 × 10^−7^, *p* = 9.074 × 10^−7^, and *p* = 1.191 × 10^−6^ for *PXDNL* variant rs80056520 by SD, CV, ASV, and VIM, *p* = 6.243 × 10^−3^ and *p* = 2.742 × 10^−3^ for *HECTD4* variant rs2074356 by SD and ASV, *p* = 5.496 × 10^−4^ and *p* = 1.299 × 10^−2^ for *CETP* variant rs2303790 by SD and ASV, respectively).

### Functional Significance of GWAS-Identified SNPs for Lipid Variability in Coronary Atherosclerosis

We evaluated the association of GWAS-derived SNPs responsible for LDL-C and HDL-C variability with coronary atherosclerosis assessed by CACS and coronary artery stenosis. Three SNPs (*APOA5* rs662799, *APOA5* rs2266788, and *PXDNL* rs80056520) were identified as being associated with significant coronary atherosclerosis after adjusting for age and sex, as shown in Table 3.

**Table 3:** Association of lipid variability SNPs with coronary atherosclerosis.

**SNPs**	**Chr**	**Position**	**Gene**		**Unadjusted OR**	**95% CI**	***P*-value**	Adjusted OR^*^	**95% CI**	***P*-value**
**CACS ≥400**										
rs80056520	8	52,466,803	PXDNL	recessive	4.174	1.096–15.900	0.036	4.057	1.009–16.309	0.049
rs2266788	11	116,660,686	APOA5	recessive	2.009	1.034–3.905	0.040	2.196	1.098–4.394	0.026
**CT stenosis ≥70%**										
rs80056520	8	52,466,803	PXDNL	recessive	9.248	1.743–49.059	0.009	9.101	1.566–52.904	0.014
rs2266788	11	116,660,686	APOA5	recessive	3.161	1.180–8.473	0.022	3.083	1.100–8.639	0.032
rs662799	11	116,663,707	APOA5	recessive	2.964	1.326–6.628	0.008	3.263	1.406–7.569	0.006

*CT, computed tomography; CI, Confidence Interval; CACS, Coronary artery calcium score; OR, odds ratio*.

* *OR adjusted for age and sex*.

Among the two genetic variants for LDL-C variability (rs2266788 and rs662799), the SNP rs2266788 in *APOA5* was significantly associated with increased risks of having CACS ≥400 (adjusted odds ratio [OR] 2.196, 95% confidence interval [CI] 1.098–4.394, *p* = 0.0262) and coronary stenosis ≥70% (adjusted OR 3.083, 95% CI 1.18–8.639, *p* = 0.0323). The SNP rs662799 of *APOA5* significantly increased the risk of having coronary stenosis ≥70% (adjusted OR 3.263 95% CI 1.406–7.569, *p* = 0.0059), but not that of having CACS ≥400.

The SNP rs80056520 in *PXDNL*, an identified genetic variant for HDL-C variability, was significantly associated with an increased risk of CACS ≥400 (adjusted OR 4.057, 95% CI 1.009–16.309, *p* = 0.0485). The SNP rs80056520 was also associated with coronary stenosis ≥70% (adjusted OR 9.101, 95% CI 1.566–52.904, *p* = 0.0139).

## Discussion

In this study, we used a GWAS to identify SNP variants associated with LDL-C and HDL-C variability in statin-naïve Korean subjects and studied their functional significance in relation with coronary atherosclerosis. We found two novel loci (*APOA5* rs662799 and *APOA5* rs2266788) associated with LDL-C variability and four novel loci (*PXDNL* rs80056520, *ALDH2* rs671, *HECTD4* rs2074356, and *CETP* rs2303790) significantly associated with HDL-C variability. Among these variant SNPs, *APOA5* rs662799 passed Bonferroni correction and was also replicated in the replication set. Moreover, we showed that three loci among the six SNP variants found were associated with significant coronary atherosclerosis, increasing the translational value of our findings, particularly in relation to clinical implications.

### Clinical Significance of Lipid Variability

Together with achieving target levels for ASCVD risk factors, maintaining consistently optimal control of risk factors with fewer fluctuations is an important aspect of ASCVD prevention. Much interest has been recently engendered about the prognostic value of intra-individual variability, including BP, glucose, and lipid profiles, which are representative risk factors for ASCVD. Mounting evidence indicates that the fluctuation of these risk factors is linked with increased risks of cardiovascular and cerebrovascular outcomes (9, 10, 19–22). When focusing on lipid variability, its role as a potential predictor of future adverse events was firstly noted in patients with known CAD (9, 10, 17). To extend these results to a general population setting, Kim et al. performed a nationwide population-based study including 3,656,648 Korean subjects (11), and showed that high lipid variability was significantly associated with adverse cardiovascular events and all-cause mortality, even after adjusting multiple traditional cardiovascular risk factors (11). The exact mechanism of poor outcomes driven by lipid variability remains unclear, but one reasonable explanation is that fluctuating lipid levels are a reflection of interruption or irregular use of statins, whose association with adverse ASCVD events is well-established. Thus, in our previous study, we excluded subjects taking statins at baseline and censored those during follow-up, to minimize the confounding effects related to statin use, such as dose change and compliance, which can directly influence lipid variability (23). In this study only including from a statin-naïve young population, high lipid variability, as opposed to abnormal lipid levels, was not associated with increased risk of MI and stroke, implying the previously reported link between lipid variability and adverse ASCVD events might be confounded by statin therapy, and ultimately raising doubt about a causal role of lipid variability in the development and prognosis of ASCVD. Another possible explanation is that lipid variability hinders lipid efflux from atheroma and consequently induce plaque progression and increase its vulnerability (24, 25). However, the biological mechanisms underlying lipid variability and the association with the fate of atherosclerosis still requires further investigation. In the present study, we have selected only statin-naïve subjects to eliminate the effect of statin exposure and showed that those with SNP variants associated with LDL-C and HDL-C variability had increased risk of CACS ≥400 and coronary stenosis ≥70%, suggesting a genetic contribution to lipid variability and its link with ASCVD.

### Genetic Variants Associated With LDL-C Variability

The two SNPs rs2266788 and rs662799 are previously described genetic variants associated with APOA5 (26–28), which are markers for classic hyperlipoproteinemia phenotypes and metabolic syndrome (29–31). Specifically, APOA5 induces lipolysis through the increase in lipoprotein lipase activity and facilitation in removal of lipoprotein particles (12), and thus genetic variants leading to dysfunctional APOA5 protein can result in dysregulation of lipolysis and lipid metabolism (32). In a meta-analysis of 91 studies including 51,868 subjects of Asian, European, and other ethnic populations, the APOA5 rs662799 SNP showed significant effects on TG, LDL-C, and HDL-C levels (33). The APOA5 rs2266788 minor allele carriage was also strongly associated with high TG and low HDL-C levels in another study from Korean population (26). The APOA5 rs662799 SNP showed a significant association with the risk for metabolic syndrome in various ethnic groups including Korean, Chinese, and Hungarian (30, 33, 34). Considering that all these findings suggested the role of two APOA5 SNPs rs2266788 and rs662799 in abnormal lipid levels, there seems to be relatively good biological plausibility for the association between these genetic variants of APOA5 and lipid variability observed in our study as compared with other genes and loci whose biological function is completely unknown. However, the role of APOA5 genetic variants in lipid variability cannot be directly extrapolated from the results supporting that in abnormal lipid levels. There is emerging evidence that the APOA5 genetic variants may contribute to ASCVD beyond their effects on lipid levels. For instance, a previous study demonstrated a strong association between rs662799 and the risk of early MI after adjusting for triglyceride levels, raising the possibility that this APOA5 SNP can affect the risk for early-onset MI beyond its known effects on lipid levels (12, 35). Our findings allow careful speculation that the APOA5 genetic variants may exert additional atherogenic effects that are mediated by lipid variability. Further studies are needed to investigate this speculation and therefore to gain better understanding of the genetic variants associated with lipid variability, beyond the genetic variants that are already known to cause abnormal lipid levels.

### Genetic Variants Associated With HDL-C Variability

Conversely, not much is known about the effect of *PXDNL* rs80056520 SNP on lipid metabolism nor coronary atherosclerosis. Peterfi et al. first discovered PXDNL as a novel peroxidase homolog and found that its expression was increased in the failing myocardium (36). Although the role of PXDNL in vascular health has not been investigated clearly peroxidase, a homolog of PXDNL, is known to promote vascular disease through oxidizing lipoproteins and uncoupling endothelial nitric oxide synthase (37). Furthermore, vascular peroxidase 1 has been suggested as having a role in the regulation of lipid homeostasis and the development of atherosclerosis, by mediating ApoE oxidation and impairing plasma lipid clearance (38). Given that PXDNL is a peroxidase homolog, it can be carefully speculated that PXDNL has a similar role in lipid metabolism and atherosclerosis. Although the exact mechanism is to be elucidated, our study demonstrated for the first time that individuals with PXDNL SNP rs80056520 had higher HDL-C variability and moreover, higher risk of having significant coronary atherosclerosis defined by CACS ≥400 and coronary stenosis ≥70%, compared with their counterparts. These findings support the possibility of a genetic predisposition both in HDL-C variability and the risk of ASCVD. Other well-known genetic variants for HDL-C levels are the mutations associated with apolipoprotein A-I, adenosine triphosphate binding cassette protein A1, lecithin cholesterol acyltransferase, lipoprotein lipase, and CETP (39). From our Korean cohort, the CETP rs2303790 SNP was significantly associated with HDL-C variability, but not with the risk of CACS ≥400 nor coronary stenosis ≥70%.

### Clinical Implications of Genetic Variants for Lipid Variability

In this study, we evaluated the possible link between potential lipid variability-related genes and coronary atherosclerosis in statin-naïve subjects, after excluding the confounding effect of lipid lowering agents. The novelty of our study relies in not only discovering genes related with lipid variability, but also showing their possible functional significance using CT findings.

With a tremendous improvement in diagnostic methods, CT-based screening for CAD has gained a significant role in detecting subclinical atherosclerosis, before clinically evident disease develops (40, 41). CACS is now regarded as an indicator of subclinical CAD, showing a strong correlation with the extent of atherosclerosis and an incremental predictive value over traditional cardiovascular risk factors. CACS reflects both the plaque burden and the severity of atherosclerotic changes in coronary arteries and is associated with adverse cardiovascular outcomes (42–44). CACS also has a clinically useful role in risk stratification, especially in intermediate risk subjects, which is recommended in current guidelines (12, 45). Intriguingly, a recent study showed that the appropriate age for initiating CAC testing was approximately 42 years for men and 58 years for women without ASCVD risk factors, but this age was shortened to 37 years for men and 50 years for women when they have diabetes (46), suggesting that the optimal timing of CACS screening differs according to the individual risk-factor profiles for premature development of atherosclerosis. However, no established framework is available to guide CAC testing to detect the earliest manifestations of CAC at young ages. Considering that genetic risk factors can be measured early in life and remain constant throughout the individual's lifetime, understanding and identifying genetic determinants of premature CAC may allow for a more individualized diagnostic approach. Our study supports this possibility by showing that *APOA5* rs2266788 related with LDL-C variability and *PXDNL* rs80056520 related with HDL-C variability may be the candidate genetic variants contributing to increased risk of CAC, independent of age and gender.

Despite the ability of CACS in clinical outcome prediction, CACS of zero still cannot guarantee the absence of significant CAD and vulnerable plaques (47), since CACS only reflects calcified plaque burden. The major advantage of CCTA is its ability to assess degree of stenosis and plaque characteristics, including not only calcified plaque, but also lipid-rich plaque, non-invasively. It is therefore not surprising that CCTA provides additional information beyond CACS, and widely used in real-world practice Also, from a previous study comparing the prognostic power of clinical, biochemical, and imaging parameters in asymptomatic Korean population, degree of coronary artery stenosis measured by CCTA was an independent predictor for adverse outcomes and was a better prognostic marker than CACS (48). However, contrast agent-related adverse effects and the relatively high cost of CCTA hampers its routine use, particularly in asymptomatic population. Again, identification of genetic risk factors for significant coronary artery stenosis may enable selection of a tailored diagnostic pathway at the individual level. Although further validation is warranted, we found that *APOA5* rs662799 was a significant genetic determinant of coronary artery stenosis ≥70%, but not of advanced CAC, raising the possibility that this genetic variant carries significant information about the risk of non-calcified plaque development and progression.

### Applying Different Metrics for Lipid Variability Measurement

Among the four different metrics of lipid variability described in the Methods, SD, and ASV were used for the main analysis in our study. Multiple metrics have been introduced to assess visit-to-visit variability, mostly regarding blood pressure variability, which include SD, SD independent of mean (SDIM), CV, ASV, range, and others. Several studies investigated the usefulness of applying different metrics and studies showed excellent interrelationships of variability metrics with one another (49, 50). Levitan et al. (50) compared visit-to-visit variability metrics for BP variability and found that most of the parameters showed a higher agreement than would be expected by chance (*p* < 0.001 for all comparisons). Notably, different variability metrics seem to convey the same information. For instance, it has been suggested that SD, SDIM and CV show nearly the same information about the variability around the mean values across visits, whereas ASV reflects the variability from one visit to the next visit. We therefore chose SD and ASV from the four different metrics of lipid variability in this study, as SD is a metric of overall variability and ASV is a metric of variability between consecutive visits. When we additionally analyzed using VIM and CV, results were similar to those using SD and ARV. *APOA5* rs662799 and *APOA5* rs2266788 were significantly related with LDL-C variability as measured by VIM and CV. *PXDNL* rs80056520 was significantly related with HDL-C variability as measured by VIM and CV.

### Limitations

First, since the number of the study population is not big enough to represent most of Korean population, our result should not be extrapolated to the general population. However, our result contains medical history and blood test results together with genetic analysis, allowing a more comprehensive evaluation of the clinical significance of genetic mutation. Second, our study was designed as a retrospective cohort study with the inherent problems, such as selection bias and unmeasured confounders, limiting the strength of our results. For example, the change in diet and physical activity can affect lipid variability, which could not be thoroughly measured in our study. Furthermore, knowing the result of CT scan *per se* may result in the change in diet and physical activity, which can in turn lead to increased lipid variability. Considering that HDL-C is known to have a strong inherited basis, although environmental factors also have a role, the confounding effects due to unmeasured variables may be relatively small when interpreting results on HDL-C variability, compared with those on LDL-C. Third, instead of taking major adverse cardiovascular events as an outcome measure, we defined study outcomes using CT findings, including coronary artery luminal stenosis and CACS, since our study population consisted of asymptomatic subjects at low risk of developing adverse clinical events. However, these imaging parameters derived from CT are increasingly being used as study endpoints for several reasons: (1) there is robust evidence that coronary artery luminal stenosis and CACS can help more accurately predict future risk of clinical ASCVD in a wide spectrum of populations, including even asymptomatic subjects without known CAD (51–55); (2) these imaging metrics are already widely used in the real-world clinical practice and their use will expand with increased availability and reliability and reduced costs, enhancing the translational applicability of research results; and (3) imaging can provide anatomic and functional information with shorter study durations and smaller sample sizes than possible with typical research assessing cardiovascular morbidity and mortality, enabling more efficient study conduct. Fourth, our study cannot determine whether high lipid variability is a disease mediator or effect moderator between SNP variants and advanced coronary atherosclerosis, for which further studies are warranted. Lastly, our data has not been validated externally in a different cohort, and should be extrapolated carefully.

## Conclusion

We found six SNP variants associated with LDL-C and HDL-C variability in statin-naïve Korean population. The *APOA5* rs662799 SNP was a significant determinant of LDL-C variability and also associated with significant coronary artery stenosis. Among other SNPs, *APOA5* rs2266788 and *PXDNL* s80056520 showed significant associations with CAC ≥400 and coronary artery stenosis ≥70%. Considering that genetic variants responsible for ASCVD, especially in association with lipid variability, have not been evaluated, our findings provide the grounds for a better understanding of potential mechanism of increased ASCVD in individuals with high lipid variability, which could lead to future mechanistic research. Further studies are also warranted to determine whether therapeutic strategies targeting lipid variability can lead to the reduced risk of advanced coronary atherosclerosis and major adverse clinical events.

## Data Availability Statement

The data analyzed in this study is subject to the following licenses/restrictions: Complete summary statistics of the GENIE cohort are not publicly available due to restrictions (institutional policy to protect the privacy of research participants), but are available from the corresponding author on reasonable request. However, all other data are contained in the article or are available upon reasonable request. Requests to access these datasets should be directed to hyoeunmd1@gmail.com or 65529@snuh.org.

## Ethics Statement

The Institutional Review Board (IRB) of the Seoul National University Hospital approved the storage of bio-specimens with written informed consent (IRB number H-1103-127-357). We retrospectively collected the clinical and genetic parameters, for which the IRB approved this study protocol (IRB number H-1803-081-930) and waived additional informed consent. This study was also performed in accordance with the Declaration of Helsinki.

## Author Contributions

J-BP, HP, and ES: conceptualization, writing—original draft, and writing—review and editing. J-BP, HP, ES, SL, S-YC, and HL: data curation. J-BP, HP, ES, J-EL, and SL: formal analysis. J-BP, HP, S-YC, HL, ES, and J-EL: investigation. J-BP, HP, ES, S-YC, HL, and SC: methodology. J-BP, HP, ES, S-YC, EC, and SC: project administration. HP, HL, S-YC, EC, and SC: resources. J-BP, HP, ES, SL, and J-EL: software. J-BP, HP, ES, EC, and SC: supervision. ES, J-EL, SL, EC, and SC: validation. ES, J-EL, and SL: visualization. All authors contributed to the article and approved the submitted version.

## Funding

This study was supported by grant no. 27-2017-0050 from the SNUH Research Fund.

## Conflict of Interest

ES, SL, and J-EL are employed by DNA Link, Inc. DNA Link, Inc. provided support in the form of salaries to these authors. There are no patents, products in development or marketed products to declare. The remaining authors declare that the research was conducted in the absence of any commercial or financial relationships that could be construed as a potential conflict of interest.

## Publisher's Note

All claims expressed in this article are solely those of the authors and do not necessarily represent those of their affiliated organizations, or those of the publisher, the editors and the reviewers. Any product that may be evaluated in this article, or claim that may be made by its manufacturer, is not guaranteed or endorsed by the publisher.
