# Corrigendum: Genetic Determinants of Visit-to-Visit Lipid Variability: Genome-Wide Association Study in Statin-Naïve Korean Population

**DOI:** 10.3389/fcvm.2022.869777

**Published:** 2022-03-01

**Authors:** Jun-Bean Park, Eunsoon Shin, Jong-Eun Lee, Seung Jae Lee, Heesun Lee, Su-Yeon Choi, Eun Kyung Choe, Seung Ho Choi, Hyo Eun Park

**Affiliations:** ^1^Division of Cardiology, Department of Internal Medicine, Seoul National University Hospital, Seoul, South Korea; ^2^DNA Link, Inc., Seoul, South Korea; ^3^Division of Cardiology, Department of Internal Medicine, Healthcare System Gangnam Center, Seoul National University Hospital, Seoul, South Korea; ^4^Department of Surgery, Healthcare System Gangnam Center, Seoul National University Hospital, Seoul, South Korea; ^5^Division of Pulmonology, Department of Internal Medicine, Healthcare System Gangnam Center, Seoul National University Hospital, Seoul, South Korea

**Keywords:** cholesterol variability, coronary artery calcium, coronary artery stenosis, genome wide association study, Apo A5

In the original article, Figures 1, 2 were published in the incorrect order. The corrected order of the figures appears below.

**Figure 1:**
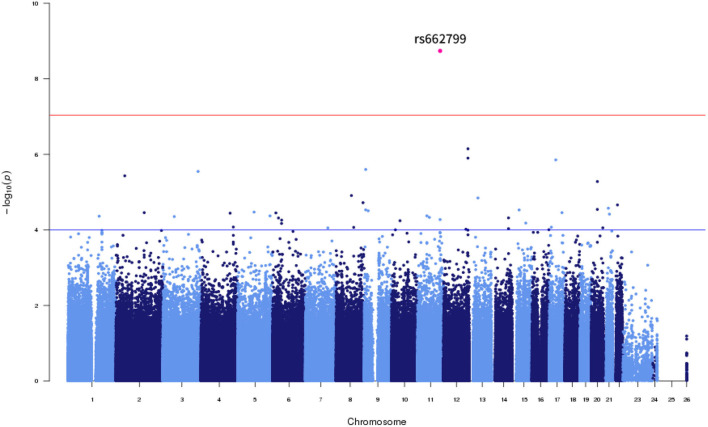
Manhattan Plot for LDL cholesterol variability by SD.

**Figure 2:**
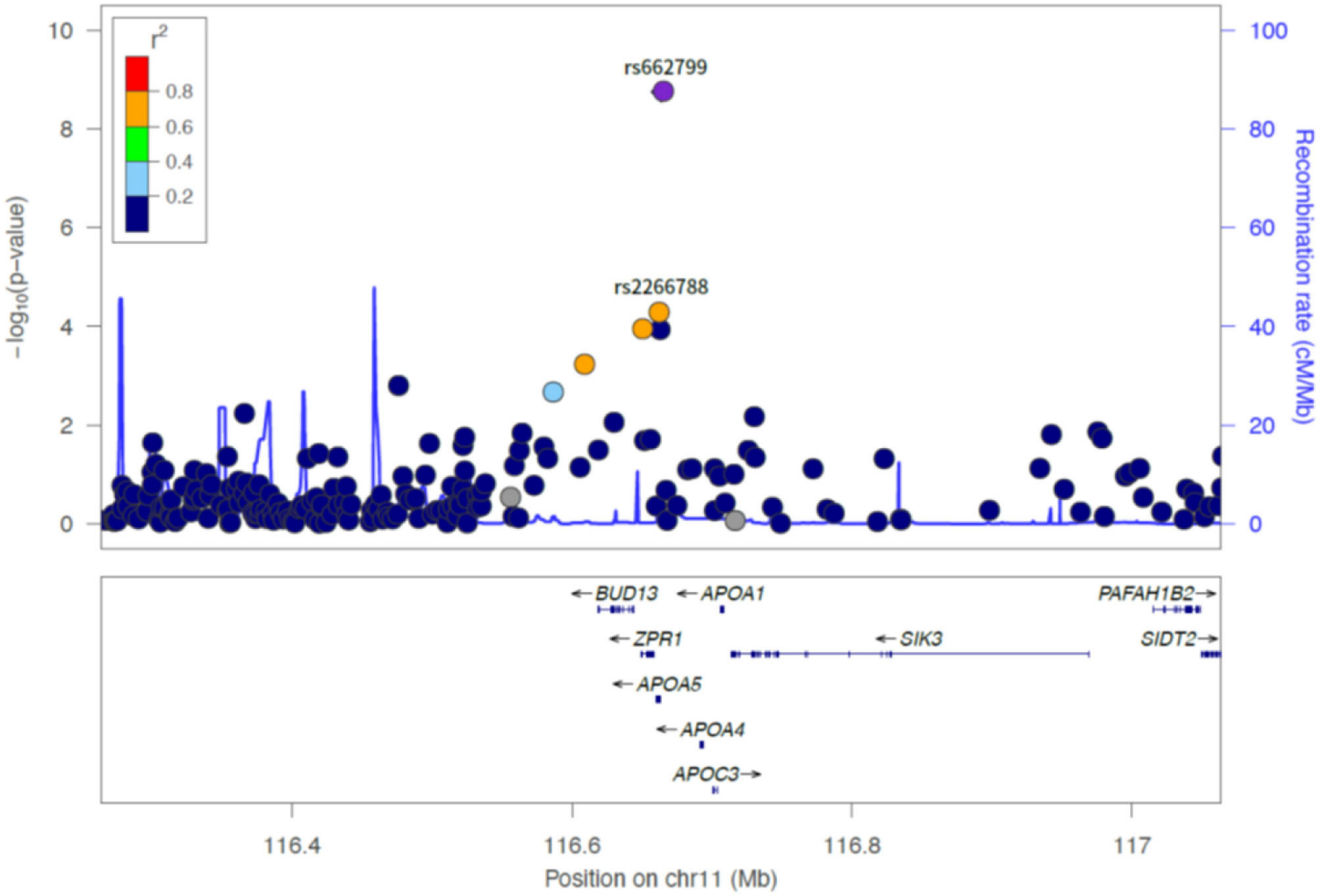
LocusZoom plot showing the region associated with LDL cholesterol variability near rs662799 (APOA5 gene).

The authors apologize for this error and state that this does not change the scientific conclusions of the article in any way. The original article has been updated.

## Publisher's Note

All claims expressed in this article are solely those of the authors and do not necessarily represent those of their affiliated organizations, or those of the publisher, the editors and the reviewers. Any product that may be evaluated in this article, or claim that may be made by its manufacturer, is not guaranteed or endorsed by the publisher.

